# Metabolic features of Gulf War illness

**DOI:** 10.1371/journal.pone.0219531

**Published:** 2019-07-26

**Authors:** Robert K. Naviaux, Jane C. Naviaux, Kefeng Li, Lin Wang, Jonathan M. Monk, A. Taylor Bright, Hayley J. Koslik, Janis B. Ritchie, Beatrice A. Golomb

**Affiliations:** 1 The Mitochondrial and Metabolic Disease Center, University of California San Diego School of Medicine, San Diego, California, United States of America; 2 Department of Medicine, Division of Medical Genetics, University of California San Diego School of Medicine, San Diego, California, United States of America; 3 Department of Pediatrics, Division of Genetics, University of California San Diego School of Medicine, San Diego, California, United States of America; 4 Department of Pathology, Division of Comparative Pathology, University of California San Diego School of Medicine, San Diego, California, United States of America; 5 Department of Neurosciences, Division of Pediatric Neurology, University of California San Diego School of Medicine, San Diego, California, United States of America; 6 Department of Medicine, Division of General Internal Medicine, University of California San Diego School of Medicine, San Diego, California, United States of America; University of California, San Francisco, UNITED STATES

## Abstract

**Background:**

More than 230,000 veterans—about 1/3 of US personnel deployed in the 1990–1991 Persian Gulf War—developed chronic, multi-symptom health problems now called “Gulf War illness” (GWI), for which mechanisms and objective diagnostic signatures continue to be sought.

**Methods:**

Targeted, broad-spectrum serum metabolomics was used to gain insights into the biology of GWI. 40 male participants, included 20 veterans who met both Kansas and CDC diagnostic criteria for GWI and 20 nonveteran controls without similar symptoms that were 1:1 matched to GWI cases by age, sex, and ethnicity. Serum samples were collected and archived at -80° C prior to testing. 358 metabolites from 46 biochemical pathways were measured by hydrophilic interaction liquid chromatography and tandem mass spectrometry.

**Results:**

Veterans with GWI, compared to healthy controls, had abnormalities in 8 of 46 biochemical pathways interrogated. Lipid abnormalities accounted for 78% of the metabolic impact. Fifteen ceramides and sphingomyelins, and four phosphatidylcholine lipids were increased. Five of the 8 pathways were shared with the previously reported metabolic phenotype of males with Myalgic Encephalomyelitis/Chronic Fatigue Syndrome (ME/CFS). However, 4 of the 5 shared pathways were regulated in opposite directions; key pathways that were up-regulated in GWI were down-regulated in ME/CFS. The single pathway regulated in the same direction was purines, which were decreased.

**Conclusions:**

Our data show that despite heterogeneous exposure histories, a metabolic phenotype of GWI was clearly distinguished from controls. Metabolomic differences between GWI and ME/CFS show that common clinical symptoms like fatigue can have different chemical mechanisms and different diagnostic implications. Larger studies will be needed to validate these findings.

## Introduction

Gulf War illness (GWI) is a complex, multi-organ system condition, affecting about 1/3 of US personnel deployed to the 1990–1991 Persian Gulf War [1], for which no single objective diagnostic test yet exists. The condition is characterized by multiple symptoms spanning a range of domains, such as fatigue and sleep disturbances, cognitive and mood changes, musculoskeletal, gastrointestinal and other symptoms. The National Academy of Medicine (NAM), previously called the Institute of Medicine (IOM), is a branch of the US National Academy of Sciences under congressional charter to provide authoritative reports on medical topics of national importance. In 2014, the NAM (under its previous name, the Institute of Medicine) summarized the case definitions that have been used for GWI [2], as listed in Box 1. Symptoms began to emerge during military service in the Gulf War from 1990–1991, and continued to accrue at elevated rates in years following deployment [3]. Of note, the ground war lasted only 4 days, many personnel never saw combat, and combat stress has consistently failed to show significance as a predictor with adjustment for other exposures [4]. What distinguished this conflict was the range of new, unique and excessive environmental and drug exposures, for some of which a dose-response relationship with illness is documented [5, 6].

**Box 1: Summary of Diagnostic Criteria for Gulf War Illness**

**Kansas Criteria** [7]: Chronic symptoms in 3 or more of the following 6 domains dating to service in 1990:

1. Fatigue and sleep problems
2. Pain symptoms
3. Neurologic, cognitive, or mood
4. Gastrointestinal symptoms
5. Respiratory symptoms
6. Skin symptoms

Requires at least 1 moderately severe symptom or ≥ 2 symptoms in a single domain for the domain to qualify. Exclusions include: cancer, diabetes, heart disease, chronic infectious disease, liver disease, lupus, multiple sclerosis, stroke, or psychiatric condition with psychosis or requiring hospitalization since 1991. Exclusions for comorbid diseases are for research purposes. Veterans with GWI are not protected from developing these conditions, and in some cases may be at elevated risk.

**CDC Criteria** [8]: At least 1 symptom in 2 of 3 categories below lasting at least 6 months:

1. Fatigue
2. Mood and cognition problems (depression, memory, sleep, or word-finding problems)
3. Musculoskeletal pain or stiffness

Numerous objective markers have been shown to be statistically altered in veterans with GWI. There is autonomic dysfunction including loss of normal heart rate variability [9, 10], increased risk of metabolic syndrome [11], increased orthostatic intolerance [10], depressed natural killer cell number and activity [12–14], increased autoantibodies [12, 15], post-exercise pain or cognitive dysfunction [16], coagulation activation [17, 18], somewhat increased inflammation [18, 19], brain imaging abnormalities [20], including effects on gray and white matter [16, 21–23], gene expression alterations [24], cognitive impairment [23, 25], mitochondrial dysfunction [26, 27], and sleep-disordered breathing abnormalities [28].

No single abnormality has been found to be universal in GWI. Individuals can achieve a similar biological response and clinical outcome in response to an insult by altering different metabolites in a pathway, with the individual profile influenced by genes and past exposures: metabolite shifts are a product of the gene-environment interaction. However, *patterns* of disturbance in functionally related pathways can constitute a biosignature that can be useful in diagnosis and in studies of pathophysiology. Metabolomics has emerged as a powerful new tool in the systems biology of complex disease [29], drug discovery, precision, and personalized medicine [30]. In this study we applied targeted, broad-spectrum metabolomics to determine if an objective chemical signature is present in veterans with GWI.

## Materials and methods

### Participants

This study was approved by the University of California, San Diego institutional review board (IRB) and Human Research Protection Program (HRPP) office under project #100959 directed by Dr. Golomb and was conducted with signed informed consent. GWI participants were 20 community-based male veterans from the San Diego area, who had been deployed to the Persian Gulf theater at any point between the dates of August 1990 and July 1991. Enrolled veterans met both CDC and Kansas symptom inclusion criteria for GWI [7, 8]. Control participants were also community-based, healthy nonveterans from the San Diego area, recruited by word-of-mouth, advertisements in local free periodicals, email list serves, and distribution of flyers. Controls were matched 1:1 to a veteran, on sex, age (within 4 years), and ethnicity (half matches accepted). Controls met neither Kansas nor CDC symptom criteria for GWI, nor had a Kansas exclusion criterion—that is, a health condition such as lupus or multiple sclerosis that could produce symptoms that might be confused for those of GWI. Signed informed consent was procured and samples were collected between October 2011 and June 2013.

### Sample collection, transport, processing, and storage

Samples were collected by venipuncture into 6 ml, red top vacutainer tubes. The samples were allowed to clot for 30 minutes at room temperature. Serum and cells were separated by centrifugation at 1500 x g for 10 minutes at room temperature. Serum was transferred to labeled cryotubes and stored at -80°C prior to analysis. Specialized sample prep with immediate deproteinization for redox reactive metabolites such as malondialdehyde and cystine/cysteine ratios was not performed.

### Metabolomics

Targeted, broad-spectrum, metabolomic analysis, covering 44 biochemical pathways, was performed by LC-MS/MS as previously described [31, 32] with minor modifications. Briefly, metabolites from 90 μl of extracted serum samples were resolved by hydrophilic interaction liquid chromatography (HILIC) on a Shodex polymer based NH_2_ HPLC column (250 mm × 2.0 mm, 4 μm, Showa Denko, USA) and Shimadzu LC-20AD UHPLC system. Mobile phase A was 95% water, 20 mM (NH_4_)_2_CO_3_, 5% acetonitrile at pH 9.8. Mobile phase B was 100% acetonitrile. The gradient was: 0–3.5 min 95% B, 3.6–8 min 85% B, 8.1–13 min 75% B, 14–30 min 0% B, 31–41 min 95% B, 41.1 min stop. The flow rate was 200 μl/min and the injection volume was 10 μl [32]. Metabolites were measured in both positive and negative ionization mode by rapid polarity switching and scheduled multiple reaction monitoring (MRM) on a SCIEX QTRAP 5500 triple quadrupole mass spectrometer (LC-MS/MS) fitted with a Turbo V electrospray ionization (ESI) source under Analyst v1.6.1 (AB Sciex) control. Chromatographic peaks are identified by comparison to stable-isotope labeled internal standards, retention time, and mass spectra as previous described [32] using MultiQuant v2.1.1 (AB Sciex), confirmed by manual inspection, and the peak areas integrated. Samples were analyzed in December 2014. The -80°C storage interval was 2.9 ± 0.3 years (mean ± SD) for the GWI samples and 2.5 ± 0.6 years for the control samples (paired ttest p = 0.003). Three hundred fifty-eight (358) of 606 targeted metabolites were measurable in all serum samples.

### Metabolic network correlation analysis

Correlation networks were constructed by calculating pair-wise Pearson and Spearman correlation coefficients for the Z-scores of all metabolites (358) in each group (GWI N = 20 and Control N = 20) separately using a custom Python script. Spearman correlations, p values, false discovery rate (FDR) [33], and Storey q values [34] are listed separately for cases and controls in S3 and S4 Tables, respectively. In S3 Fig the nodes are colored by metabolic subsystem. The networks are visualized using d3 software available at: https://d3js.org/.

### Statistical analysis

Participant demographic and health data were compared using paired t-tests or Wilcoxon paired ranked sum testing for continuous data, and Fisher’s exact test for categorical data. Metabolomic data were log-transformed, scaled by control standard deviations, and the resulting Z-scores $\left({\text{X}}_{\text{case}}-{\bar{\text{X}}}_{\text{control}}\right)/{\text{SD}}_{\text{control}}$) analyzed by multivariate partial least squares discriminant analysis (PLSDA), with pairwise comparisons and post hoc correction for multiple hypothesis testing using Fisher’s least significant difference method in MetaboAnalyst (www.metaboanalyst.ca) [35, 36], or the false discovery rate (FDR) method of Benjamini and Hochberg [33]. Q values were by the method of Storey [34]. Metabolites with variable importance in projection (VIP) scores determined by PLSDA that were greater than 1.5 and Mann-Whitney U test p values ≤ 0.05 were considered significant. Significant metabolites were grouped into pathways and their VIP scores summed to determine the rank-ordered significance of each biochemical pathway. Random forest [37], K-means, and k-nearest neighbor (kNN) clustering were used to identify metabolite groups that contributed in different ways to the discrimination of GWI and controls. Classifiers of 1–6 metabolites were selected and tested for diagnostic accuracy using area under the receiver operator characteristic (AUROC) curve analysis. Sample size calculations for future studies were performed using the following parameters: two-sided α = 0.05, β = 0.2 (power = 0.8), and an observed effect size 0.51 to calculate the number of cases and controls needed to select 6 metabolites in a multiple regression model. Classifiers were validated within sample using repeated double cross validation (rdCV) [38], with bootstrapping 100 times to test random subsamples of 2/3 in and 1/3 out, and by permutation analysis [39]. Results were organized into biochemical pathways and visualized in Cytoscape version 3.4.0. Statistical methods were implemented in Stata (Stata/SE12.1, StataCorp, College Station, TX), Prism (Prism 6, GraphPad Software, La Jolla, CA), Python, or R. Metabolomic pathway abnormalities in GWI were compared to previously reported findings for myalgic encephalitis/ chronic fatigue syndrome (ME/CFS) [31].

## Results

### Participant characteristics

Table 1 shows participant characteristics. All participants were male. Mean age at sample procurement was 49 ± 1.8 (mean ± SEM) years. No significant differences in age, marital status, ethnicity, or education were observed between groups. On average, GWI veterans were slightly heavier than controls; BMI = 30.4 ± 3.8 in the GWI group and 27.9 ± 3.6 in controls; p = 0.04. This is consistent with reports of increased weight gain in Gulf-deployed veterans relative to nondeployed controls [8, 40, 41]. As outlined in Table 2, affected veterans had twice as many major surgeries, 12-times more intercurrent illnesses, 4-times more infections, 3.5-times more hospitalizations, 6-times more medications, and 4-times more comorbid conditions compared to controls. Similar findings in GWI have been previously reported [40–45].

**Table 1:** Participant demographics.

	Gulf War illness (n = 20)	Controls (n = 20)	p
**Age in years ± SEM (range)**	49 ± 1.8 (41–65)	48 ± 1.9 (39–66)	ns
**% Male**	100	100	ns
**% Married**	65	35	ns
**Ethnicity (%)**			
Caucasian	55	55	ns
Hispanic	15	15	ns
African-American	20	20	ns
Asian	5	5	ns
Native American	5	5	ns
**Education (%)**			
High school graduate	5	5	ns
Technical school	0	5	ns
Associate’s Degree	40	40	ns
Bachelor’s Degree	30	40	ns
Master’s Degree	20	5	ns
Doctorate	5	5	ns

**Table 2:** Participant health data.

	Gulf War illness Mean ± SEM (Range)	Controls Mean ± SEM (Range)	p
**BMI (kg/m**^**2**^**)**	30.4 ± 3.8 (24–40)	27.9 ± 3.6 (21–38)	0.04
**# Surgeries**	2.5 ± 0.46 (0–8)	1.25 ± 0.26 (0–4)	0.02
**# Major Illnesses**	0.6 ± 0.27 (0–5)	0.05 ± 0.05 (0–1)	0.05
**# Major Infections**	0.4 ± 0.12 (0–1)	0.1 ± 0.07 (0–1)	0.03
**# Hospitalizations**	1.75 ± 0.44 (0–7)	0.5 ± 0.22 (0–3)	0.01
**# Comorbid Conditions**	1.25 ± 0.33 (0–5)	0.3 ± 0.13 (0–2)	0.009
**# Medications**	8.2 ± 1.5 (1–24)	1.4 ± 0.34 (0–5)	<0.0001

Medication use was heterogeneous. Over 100 different medications were used by the GWI participants and 18 by controls (Table 2; p < 0.0001). However, no single medication was taken by more than 6 participants out of 20 in each group. The most commonly used supplement or medication in the GWI group was fish oil, cod liver oil, or omega 3 supplement, which was used by 6 participants in the GWI group and 4 participants in the control group. As a group of symptom-related drugs, 9 different antihypertensive medications were taken by 16 of 20 GWI participants and 3 controls (p < 0.0001). Baby aspirin, ibuprofen, Tylenol or other non-steroidal anti-inflammatory drugs (NSAIDS) were used by 10 GWI participants and 3 controls (p < 0.05). No participants were taking an oral anti-inflammatory steroid such as prednisone. Although there were expected differences in medication use between GWI and controls, the metabolomic profile described below was not significantly correlated with any of the observed medication uses and none is known to produce a pattern of increased phospholipids and sphingolipids found.

### Metabolomics pathway overview

**Fig 1:**
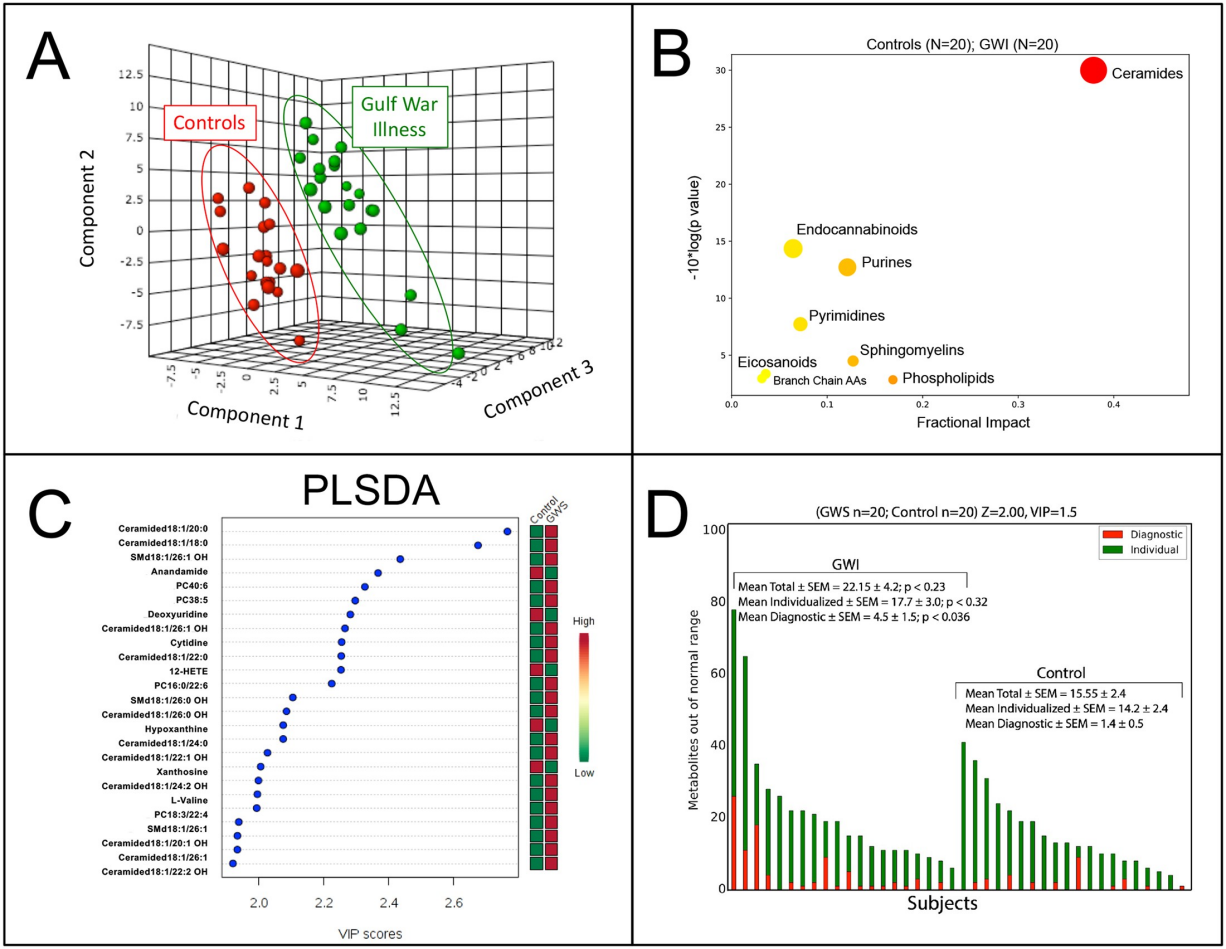
Metabolite and biochemical pathway abnormalities in Gulf War illness. **A**. Multivariate metabolomic discrimination of GWI from controls. (n = 20 males with GWI; 20 male controls). PLSDA: partial least squares discriminant analysis. **B**. Pathway bubble plot indicating the fractional metabolic impact. **C**. Rank order of the top 25 discriminating metabolites by multivariate variable importance in projection (VIP) scores. **D**. Individual vs Diagnostic Metabolite Abnormalities. Diagnostic metabolites (blue) were defined as having VIP scores of ≥ 1.5 and a Z-score of ≥ 2.0 or ≤ -2.0 in the same direction, above or below the control mean, as found by multivariate PLSDA. Individualized abnormalities (yellow) met the Z-score criterion but were not significant by VIP score and were not diagnostic for GWI.

Multivariate analysis of the metabolomic profile of clearly discriminated veterans with GWI from healthy controls (Fig 1). Significant metabolites (S1 Table) were analyzed by pathways ranked according to their metabolic impact (Table 3, Fig 1B, S1 Table). Lipids dominated the observed differences in veterans with GWI, contributing 78% of the metabolic impact. Important roles were also played by purines and pyrimidines, which contributed 19% of the metabolic impact (Table 3, Fig 1B and 1C, S1 Table).

**Table 3:** Biochemical pathways disturbed in Gulf War illness.

No.	Pathway Name	Measured Metabolites in the Pathway (N)	Expected Pathway Proportion (P = N/358)	Expected Hits in Sample of 30 (P * 30)	Observed Hits in the Top 30 Metabolites	Fold Enrichment (Obs/Exp)	Impact (Sum VIP Score)	Fraction of Impact Explained (% of 63.0)	Increased	Decreased
1	Ceramide Metabolism*	31	0.09	2.6	11	4.2	23.9	38%	11	0
2	Phospholipid Metabolism*	56	0.16	4.7	5	1.1	10.6	17%	4	1
3	Sphingomyelin Metabolism*	36	0.10	3.0	4	1.3	8.0	13%	4	0
4	Purine Metabolism	18	0.05	1.5	4	2.7	7.6	12%	0	4
5	Pyrimidine Metabolism	9	0.03	0.8	2	2.7	4.5	7%	1	1
6	Endocannabinoid Metabolism*	4	0.01	0.3	2	6.0	4.1	6%	0	2
7	Eicosanoid and Resolvins*	7	0.02	0.6	1	1.7	2.3	4%	0	1
8	Branch Chain Amino Acids	8	0.02	0.7	1	1.5	2.0	3%	1	0
								**Subtotal**:	21	9
								**Total**:	30	

*Lipid pathways.

### Ceramides and sphingomyelins were increased

The three most-altered biochemical pathways were ceramides, sphingomyelins, and phosphatidylcholines. These three pathways accounted for 68% of the metabolic impact (Table 3). In each case, measured pathway products were increased in veterans with GWI compared to controls. Ceramides and sphingomyelins accounted for 38% and 13% of the impact, respectively. Eleven ceramides were increased an average of 42% ± 15% (mean ± SEM). This corresponded to a mean ceramide Z-score = +0.80 ± 0.1 (SD) (S1 Table).

### Phospholipids were increased

Phospholipids accounted for 17% of the metabolic impact. Four of the five phosphoglycerolipids that were changed were phosphatidylcholine (PC) lipids that were increased by an average of 20% ± 4% compared to controls. This corresponded to a mean PC lipid Z-score of +0.78 ± 0.05. One phosphatidylserine lipid (PS 18:0/18:1) was decreased by 22% (Z-score = -0.66) in veterans with GWI (S1 Table).

### Purines were decreased

Four purine metabolites were decreased in veterans with GWI and contributed to 12% of the metabolic impact (Table 3). Inosine was decreased by 26% (Z = -0.61). Hypoxanthine was decreased by 20% (Z = -0.84). Guanosine was decreased by 30% (Z = -0.74), and its oxidative deamination product, xanthosine was decreased by 20% (Z = -0.59, S1 Table).

### Pyrimidine metabolism was changed

Pyrimidines accounted for 7% of the metabolic impact (Table 3). Deoxyuridine was decreased by 18% (Z = -0.88) in veterans with GWI. Cytidine was increased by 41% (Z = +0.89, S1 Table).

### Endocannabinoids were decreased

One endocannabinoid, anandamide, and an endocannabinoid analog N-oleoylethanolamine (OEA) were decreased in veterans with GWI and accounted for 6% of the metabolic impact. Anandamide was decreased by 18% (Z = -0.84, S1 Table), and contributed 3.5% of the metabolic impact (Table 3, VIP = 2.3). OEA was decreased by 16% (Z = -0.60, S1 Table), and contributed 2.5% of the impact (Table 3, VIP = 1.7). The other assessed endocannabinoids and analogs, 2-arachidonoyl glycerol (2-AG), and palmitoylethanolamide (PEA), were unchanged.

### 12-Hydroxyeicosatetraenoic acid (12-HETE) was decreased

12-HETE in veterans with GWI was by decreased by 63% (Z = -0.84, S1 Table), and contributed 4% of the metabolic impact (Table 3, VIP = 2.2).

### Valine was increased

Valine was increased 14% in GWI (Z = +0.64, S1 Table) and contributed 3% of the metabolic impact (Table 3, VIP = 1.96).

### Acyl-carnitines, as a group, were increased

Although no single acyl-carnitine was significantly different in the group analysis of GWI and controls, all 18 acyl-carnitine species measured with VIP scores > 0.5 were increased by an average of 14% (p < 0.001; mean Z = +0.89 ± 0.06, S1 Fig, S2 Table). Unesterified L-carnitine was unchanged. The increase in blood acyl-carnitine species containing 2–16 carbon fatty acids is consistent with a decrease in short-, medium-, and long-chain fatty acid beta oxidation, which occurs in mitochondria [46].

### GWI-associated vs individualized metabolic variations

On average, only about 20% (4.5 of 22.1 = 20%; p = 0.036) of the metabolic abnormalities found in each participant contributed to the profile of abnormalities found in veterans with GWI (Fig 1D). About 80% (17.7 of 22.1) of the metabolic changes found in each affected veteran were specific to the individual, reflecting the unique features of their particular genes, history, and illness that did not contribute to the shared metabolomic signature of GWI. The predominance of metabolic abnormalities that are specific to the individual has been observed in other metabolomic studies of complex chronic disease [31].

### Assessment of metabolomics as a diagnostic tool in Gulf War illness

**Fig 2:**
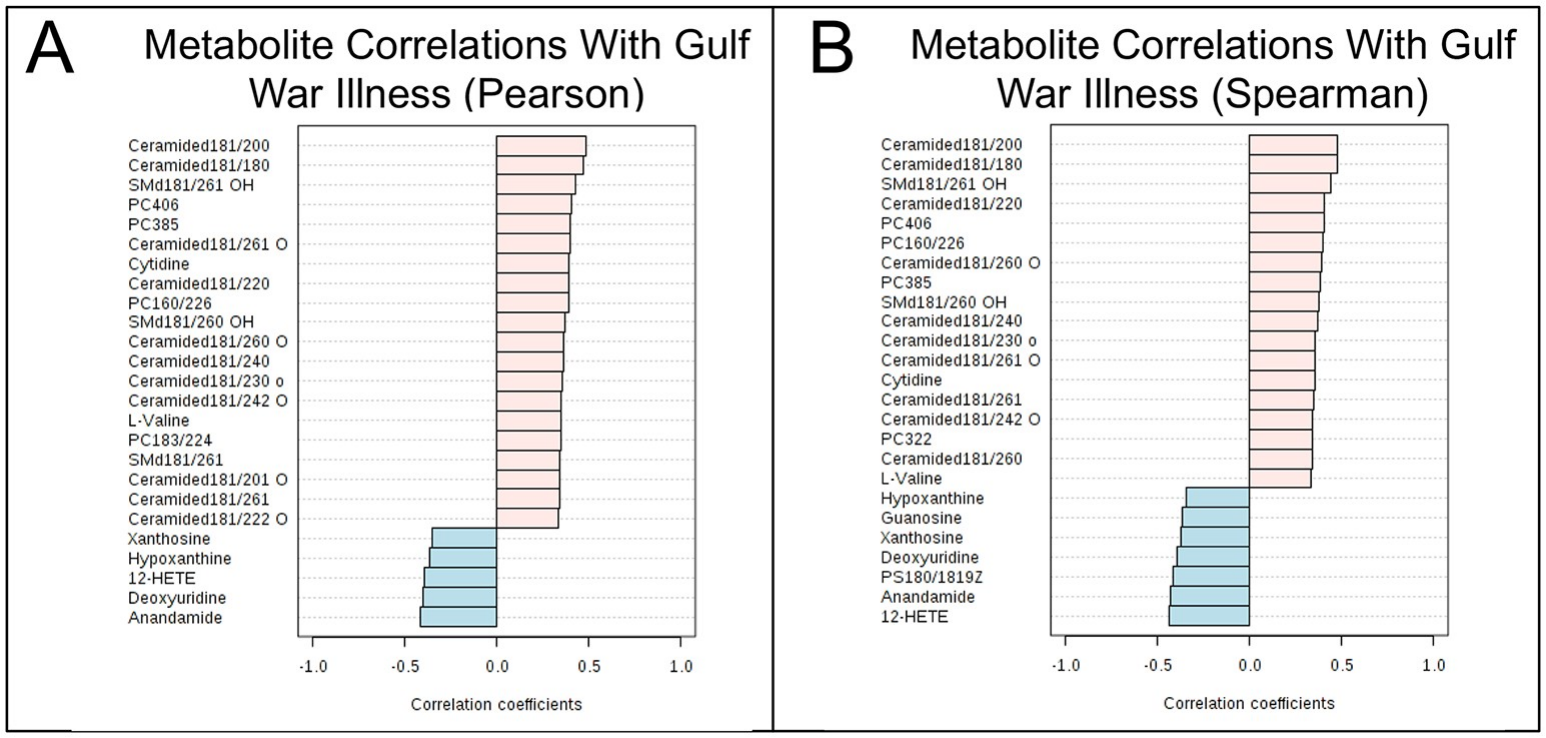
Top 25 metabolites most correlated with Gulf War illness vs healthy civilian controls. **A**. Ranked by parametric Pearson r correlation. **B**. Ranked by non-parametric Spearman rank correlation. Pink bars represent metabolites that were increased in GWI. Blue bars represent metabolites that were decreased in GWI. Pairwise correlations were based on z-scores.

**Fig 3:**
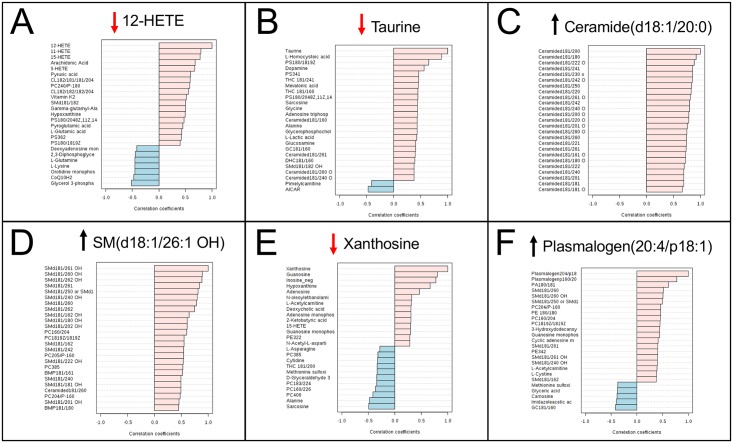
Intermetabolome correlations of the 6 metabolites selected as a classifier for the discrimination of Gulf War illness from healthy civilian controls. **A**. 12-HETE, **B**. Taurine, **C**. Ceramide(d18:1/20:0), **D**. Sphingomyelin SM(d18:1/26:1 OH), **E**. Xanthosine, **F**. Plasmalogen (20:4/p18:1). Black arrows indicate that the metabolite was increased in GWI. Red arrows indicate that the metabolite was decreased in GWI.

The top 25 metabolites that were most correlated by univariate analysis with a diagnosis of GWI are shown in Fig 2. Area under the receiver operator characteristic (AUROC) curve analysis was used to test diagnostic performance of metabolic classifiers consisting of 1–6 metabolites (Table 4). All possible combinations were not tested exhaustively. Many other sets of 1–6 metabolites may perform equally well or better. Two metabolites were included in the 6-member classifier that did not meet statistical criteria for inclusion among the top 30 metabolites (S1 Table). These were plasmalogen (20:4/p18:1) and taurine (Table 4). This plasmalogen was increased 19% (Z = +0.47; U test p = 0.07; FDR = 0.66) in GWI. Plasmalogens are specialized phospholipids made by the ER, mitochondria, and peroxisomes that are enriched in aerobic tissues like the heart and brain [47]. Taurine is an amino sulfonic acid derived from cysteine [48]. In the brain, taurine regulates cell volume and protects neurons and mitochondria against excitotoxicity [49]. Taurine was ranked #84 of 358 metabolites by PLSDA analysis, but was ranked #2 by random forest analysis. Taurine was decreased 13% (Z = -0.51) in GWI compared to controls (VIP = 1.2; U test p = 0.08; FDR = 0.79) (Fig 3). Classifiers using the sets of 4–6 metabolites illustrated in Table 4 were about 93% accurate (range = 92%-94%) by ROC curve analysis, and had permutation p values ≤ 0.004, and cross validation scores of about 83% (range = 82%-84%). Future studies will be needed to rule out overfitting, and to assess whether these 6 metabolites are robustly diagnostic in larger cohorts of GWI.

**Table 4:** Performance of metabolomics as a diagnostic tool in Gulf War illness.

No. of Analytes	Classifier	2 x 2 Contingency Table Analysis				AUROC Performance^1^		Validation	
		False Negatives/ True Positive	False Positives/ True Negatives	Sensitivity (95% CI)	Specificity (95% CI)	Accuracy (area under the curve)	95% CI	rdCV^1^	p^2^
1	12-HETE	8/12	8/12	0.60 (0.39–0.78)	0.60 (0.39–0.78)	0.69	0.43–0.90	0.624	ns
2	12-HETE, Taurine	6/14	7/13	0.70 (0.48–0.85)	0.65 (0.43–0.82)	0.72	0.47–0.93	0.658	0.088
3	12-HETE, Taurine, Ceramide(d18:1/20:0)	5/15	3/17	0.75 (0.53–0.89)	0.85 (0.64–0.95)	0.84	0.65–0.98	0.764	0.02
4	12-HETE, Taurine, Ceramide(d18:1/20:0), SM(d18:1/26:1 OH)	3/17	3/17	0.85 (0.64–0.95)	0.85 (0.64–0.95)	0.92	0.72–1.0	0.832	0.002
5	12-HETE, Taurine, Ceramide(d18:1/20:0), SM(d18:1/26:1 OH), Xanthosine	2/18	3/17	0.90 (0.7–0.98)	0.85 (0.64–0.95)	0.93	0.76–1.0	0.824	0.003
6	12-HETE, Taurine, Ceramide(d18:1/20:0), SM(d18:1/26:1 OH) Xanthosine, Plasmalogen(20:4/p18:1)	1/19	2/18	0.95 (0.76–1.0)	0.90 (0.7–0.98)	0.94	0.79–1.0	0.836	0.004

^1^ Area under the receiver operator characteristic (AUROC) curve and repeated double cross validation (rdCV) results were calculated by random forest analysis and bootstrap resampling x 100.

^2^ Empirical p value after 1000 permutations. The direction and magnitude change of the metabolites used in the classifiers are discussed in the text, illustrated in Fig 3, and listed in S1 Table.

## Discussion

Veterans with GWI had objective chemical abnormalities that distinguished them from controls. Five of the top 8 most disturbed metabolic pathways in GWI in this study involved lipid metabolism and accounted for 78% of the metabolomic impact (Table 3). Sphingolipids, phospholipids, endocannabinoids, and eicosanoid metabolism were each altered in veterans with GWI (Table 3, S1 Table). Phosphatidylcholine (PC) lipids are the principal component of mammalian membranes [50], are used as a source of phosphorylcholine for sphingomyelin synthesis [51], and can be recycled as a source of choline and betaine [52]. New synthesis of PC lipids is required for cell growth/differentiation or cell replacement in settings of high turnover [50, 53]. Other significant pathway disturbances involved purines, pyrimidines, and valine metabolism. The pathways that were altered in GWI are known to play important roles in the regulation of cell membrane functions, signal transduction, redox status, mitochondrial function, cellular energy production, apoptosis, immunity, inflammation, and the cell danger response [54].

Increased serum ceramides accounted for 38% of the metabolomic impact. Both ceramides and their salvage precursor sphingomyelins (SMs) were increased. In serum, sphingomyelin and ceramide are present in exosomes and extracellular vesicles that are released from cells exposed to toxins or insults of many different kinds [55], and in HDL and LDL lipoprotein particles under baseline conditions [56]. Ceramides are well known to inhibit mitochondrial function and can induce [57] and regulate [58] apoptosis, and promote inflammation [59]. Ceramides are also known to stimulate cellular defense mechanisms through TLR4 signaling [60], and stimulate LC3B-II dependent mitophagy [61]. Increased pro-inflammatory signaling through ceramides and decreased anti-inflammatory signaling through anandamide provide one potential metabolic basis for the low-level chronic inflammation that has been reported in GWI [19]. The pattern of increased ceramides in veterans from other wars associated with post-traumatic stress disorder (PTSD) has also been described [62]. This is consistent with the growing understanding that serine-based sphingolipid abnormalities may represent a fundamental biochemical response to infection, cell stress, or injury [63]. In contrast, glycerol-based phosphoglycerolipids were regulated differently in GWI, PTSD, and traumatic brain injury (TBI). For example, phosphatidylcholine (PC) lipids were decreased in PTSD and TBI [64] and increased in GWI (Table 3).

### Metabolic networks

Many of the metabolites that contributed to the diagnostic classifiers for GWI were found to be highly correlated with other metabolites that made up the measured metabolome (Figs 2 and 3, S3 Fig, S3 and S4 Tables). For example, an increase in ceramide d18:1/20:0 was positively correlated with 24 other ceramides (Fig 3C). In the case of the purine xanthosine, there were positive and negative correlations with both purine and non-purine metabolites (Fig 3E). In addition to the role a metabolite plays in cellular biochemistry, there are additional connections in the metabolome that arise in part because many metabolites also have a signaling role. Many metabolites bind to G-protein coupled and other receptors, and to cellular control proteins like sirtuins, CLOCK:BMAL [65], mTOR, and AMPK that alter gene expression, which in turn influence metabolism and the healing cycle [66]. Spearman correlation analysis identified 902 metabolite pairs with an FDR ≤ 0.05 in GWI (Table 5) and 589 metabolite pairs under this false discovery rate in controls (Table 5). Such connections define a resilient network or interactome that may resist external perturbation from many dietary factors, drugs, and supplements, and can remain as a feature of chronic illness for years [67]. There are 53% more significant metabolite pair correlations in GWI than in controls (902 ÷ 589 = 1.53; of 63,903 unique pairwise correlations for 358 x 358 metabolites; p < 0.0001, Fisher’s exact test). Such an extensively coupled metabolic network may arise from correlated adaptations to the environmental exposures that may have originally triggered the illness. Such adaptations may, for instance, permit earlier sensory and/or affective responses to lower doses of a given environmental trigger, permitting earlier evasive action. However, the more tightly coupled metabolic network may come at the cost of increased autonomic, immune, cardiovascular, sensory, or responses to exposure levels of environmental triggers that fail to produce responses in individuals with a more loosely coupled metabolic network.

**Table 5:** Metabolite correlation network analysis.

Parameter	GWI	Controls	P value
Total possible, unique pairwise correlations*	63,903	63,903	--
Significantly correlated metabolite pairs (FDR < 0.05)	902	589	<0.0001
Positively correlated metabolite pairs (FDR < 0.05)	835	541	ns
Negatively correlated metabolite pairs (FDR < 0.05)	67	48	ns

*By Spearman non-parametric correlation analysis. For a matrix of 358 metabolites: N = ((358 x 358)- 358)/2 = 63,903.

### Relative concentration differences in Mendelian and non-Mendelian disorders

In contrast to Mendelian disorders that cause inborn errors in metabolism and can lead to large changes of 3 to over 30-fold increases or decreases in metabolite concentrations [68, 69], the changes found in chronic complex, non-Mendelian disorders like GWI are smaller; often just ± 20% to 2-fold compared to healthy controls [31]. The average differences for the top 30 most significant metabolites found in the current study were -26% (median Z = -0.73; interquartile range = -0.61 to -0.88) for the 9 metabolites that were decreased and +34% (median Z = +0.78; IQR = +0.70 to +0.85) for the 21 that were increased (S1 Table). This small magnitude of difference for any single metabolite cannot usually be proven to be the “cause” of any particular symptom. However, in each case, the measured metabolic abnormality represents a feature of a metabolic network that can be a stable feature of chronic illness for years, and may respond dynamically to resist many external perturbations (Fig 3, S3 Fig, S3 and S4 Tables). Indeed, some of the observed metabolic changes are compensatory—they act to oppose a problem, and are negatively correlated with clinical symptom severity. Attempts to normalize one of these compensatory metabolic changes can worsen symptoms. An improved understanding of the signaling pathways that regulate the dynamics of metabolic networks as a therapeutic target has led to a deeper understanding of pathogenesis and to new approaches to treatment [66, 67, 70].

### Similarities and contrasts with metabolomic features of chronic fatigue syndrome

**Fig 4:**
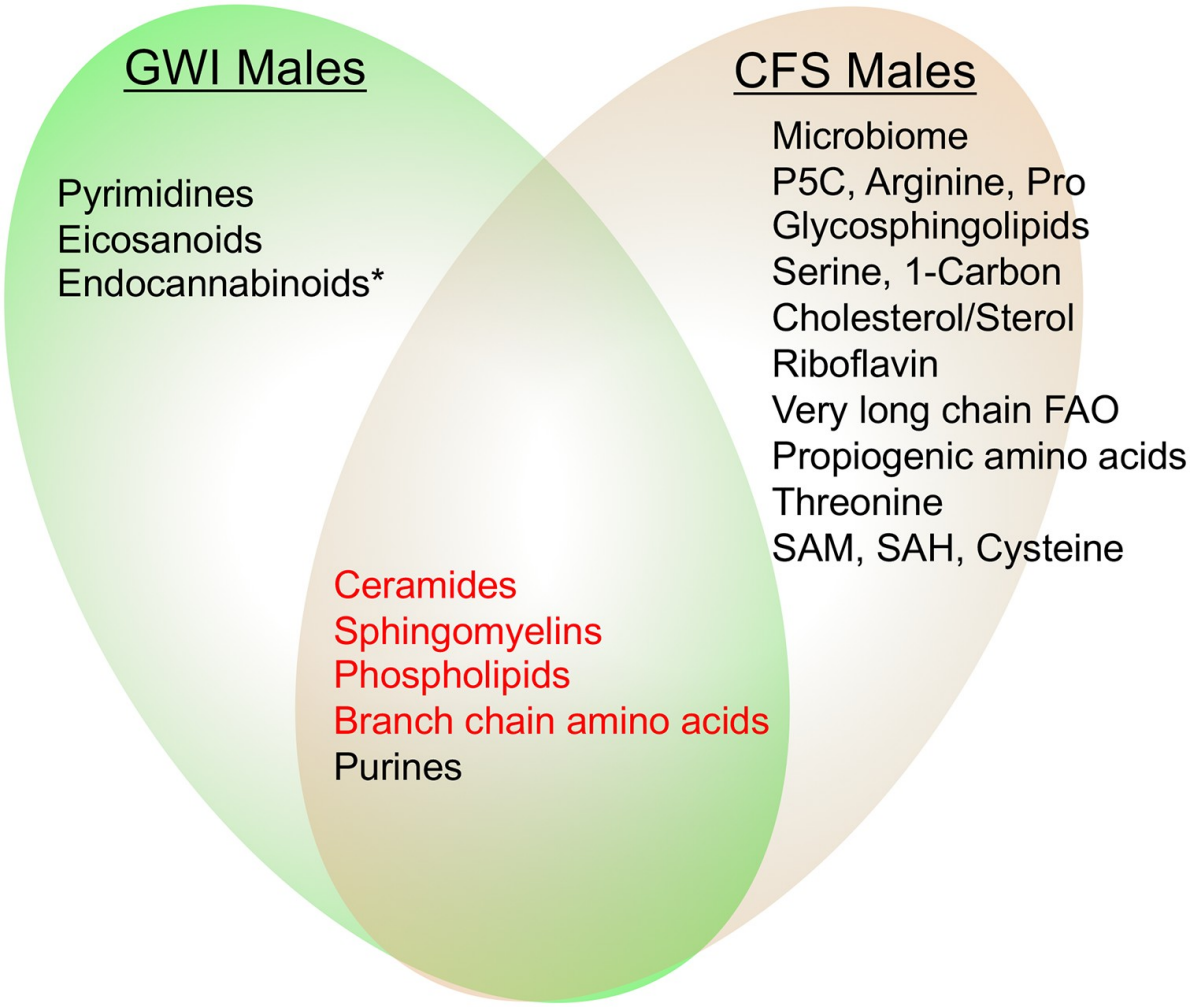
Metabolic similarities and differences between Gulf War illness and chronic fatigue syndrome. Four of five pathways shared by males with GWI and CFS were regulated in opposite directions (red font). Only purines were regulated in the same direction—decreased in both GWI and CFS. *One GWI pathway (endocannabinoids) was similarly decreased in females with CFS, but not in males with CFS [31].

Five of the 8 pathways that were disturbed in males with GWI have been previously reported to be abnormal in males with ME/CFS [31] (Fig 4). ME/CFS has been reported to be an energy reallocation program and dauer-like multisystem disease that can be triggered by a variety of infectious and environmental agents and injuries [31]. Others have interpreted the bioenergetics of ME/CFS as conserved energy use in the face of reduced energy supply from mitochondria [71]. ME/CFS shares some clinical symptoms with GWI. Shared symptoms include fatigue, sleep problems, and cognitive impairment. The prevalence of CFS is elevated in veterans with GWI [72], but the disease characteristics are reported to differ from CFS in civilians [5, 73]. For example, a contrasting symptom is post-exertional malaise, which is a defining symptom in ME/CFS [74], but is not defining or prominent in GWI. When specific metabolites were compared, we found that veterans with GWI and patients with ME/CFS showed the opposite metabolic abnormalities in 4 of 5 shared pathways (Fig 4 red shaded pathways, Table 6) [2, 74]. The only pathway that was regulated in the same direction (down in both) was purine metabolism (Tables 3 and 6, Fig 4). In addition to their broad intracellular roles as intermediates and energy carriers for metabolism, extracellular purines and pyrimidines play a critical role in purinergic signaling for regulation of energy homeostasis [75], the control of chronic pain and inflammation [76], and regulate healing [66]. Altered plasma purine pools may reflect systemic alternations in purinergic signaling [77] associated with mitochondrial metabolism, healing, and the cell danger response [54, 66, 78]

**Table 6:** Metabolic features of Gulf War illness and chronic fatigue syndrome.

Metabolic Pathway	Gulf War Illness (males)	Chronic Fatigue Syndrome (males) [31]
Ceramides and Sphingomyelins	↑	↓
Phospholipids	↑	↓
Cardiolipins	↓	↑
Purines (Xno, Ino, Hx)	↓	↓^1^
Endocannabinoids	↓	↓
HETEs, eicosanoids	↓	↓
Pyrimidines (Cytidine)	↑	Unchanged
Valine	↑	Unchanged or ↓
Arginine	Unchanged	↑
Uric acid	Unchanged	↓
Acyl-carnitines	Unchanged or ↑	Unchanged or ↓

^1^Although Xno, Ino, and Hx (xanthosine, inosine, and hypoxanthine) were unchanged in males with CFS, two other purines, uric acid and deoxyguanosine, were decreased.

### Lipidomic findings support and extend findings from mouse and rat models of GWI

Animal models of GWI have been produced by exposure to pyridostigmine bromide and permethrin, with or without N,N-diethyl-m-toluamide (DEET), seeking to capture the effect of exposure mixtures experienced in the Persian Gulf theater. A recent plasma lipidomic analysis in rat and mouse models, and 22 veterans with GWI showed elevations in phospholipids [79]. Elevated serum ceramide and sphingomyelin species in veterans with GWI represents a new finding (Table 3, S1 Table, S1 Fig). Increased sphingomyelin species, which are an important reservoir of choline for acetylcholine synthesis and cholinergic signaling, have also been reported in the brains of mouse models of GWI [80]. Amyotrophic lateral sclerosis, a condition that was elevated in Gulf War veterans in the early years after the conflict [81], has recently been found to have some metabolomic abnormalities that overlap with GWI, including elevations in phosphatidylcholines and sphingomyelins [82].

### Diagnostic utility

No single measured metabolite was diagnostic for GWI. However, a diagnostic pattern of GWI was recognizable as an increase in multiple ceramides, sphingomyelins, phosphatidylcholine lipids, and acyl-carnitines, associated with a decrease in purine nucleosides, and certain endocannabinoids. This pattern of abnormalities was diagnostic (Tables 2 and 3, Fig 2)**.**

### Study limitations

Limitations of the current study include its small size of 20 subjects per group. The small study size limited the overall statistical power and kept the mean false discovery rate for the 30 significant metabolites to 0.45 (β range = 0.38–0.66) despite mean p values of < 0.03 and multivariate VIP scores ≥ 1.5 (S1 Table). Power analysis based on the mean effect size of 0.51 observed in this study, a two-sided α = 0.05 and β = 0.2, predicts that a sample size of 35 cases and 35 controls would be needed in future studies to identify 6 metabolites as a classifier in a multiple regression model. Sample sizes might be reduced to about 25–30 with the same power by using plasma instead of serum because of improved sample processing control [83]. This study was restricted to males, and sex differences are well known in metabolomic studies [31]. Future studies should include, or focus on, females with GWI, to identify shared and disparate metabolic features.

This study used nonveteran controls. This choice was made in part because many Gulf War veterans who were not initially diagnosed with GWI, continued to accrue symptoms and newly meet GWI diagnostic criteria in the years after the war [3]. Each choice of a control population will have attendant pros and cons. In addition, metabolomics studies regularly benefit from a validated quality of life or clinical severity score that permits the change in specific metabolites to be correlated with symptom severity or functional performance [31, 84]. Finally, there was a small but statistically significant, 5-month difference between the -80°C storage time of GWI samples (2.9 ± 0.3 years) vs control samples (2.5 ± 0.6 years). Although a systematic study on the stability of all the metabolites measured by metabolomics has not yet been published, one study has shown that polyunsaturated fatty acids in serum phospholipids were stable for at least 8–10 years at -80°C [85]. We consider it unlikely that a 5-month difference in storage time after nearly 3 years at -80°C would systematically change more than a few of the 30 lipids, purines, and pyrimidines identified in this study. In unpublished studies on the effect of -80°C storage time on the metabolomic profile of plasma samples, we found that except for a short list of reactive metabolites that change within the first few months of storage then stabilize, only about 1–2% of the metabolites change their apparent concentration for each year of storage out of more than 400 we measured in plasma (RKN, unpublished data).

### General caveats for metabolomics studies

Not every metabolite can be measured with a single mass spectrometry method. Some metabolites that are abundant inside cells may be undetectable in plasma. It is possible that unmeasured metabolites, cytokines, or cellular differences would add further to diagnostic accuracy, or offer added insights into mechanism of disease in GWI. In characterizing the roles for different metabolites, not all known roles have been cited, those that are cited need not be relevant to GWI, and scientific understanding of the roles each metabolite plays continues to evolve. Although we applied the usual statistical validation methods in omics studies such as repeated double cross validation and permutation analysis [38], no metabolomic signature of a disease can be considered confirmed until it has been validated in geographically independent cohorts of the same and related disorders. Future studies will be required to test the generalizability of specific metabolic classifiers in other GWI cohorts.

### Implications for differential diagnosis, pathogenesis, and treatment

The finding of metabolic pathways that are regulated in opposite directions in GWI and CFS (Fig 4, Table 3), and the lipidomic differences that distinguish GWI from PTSD and TBI suggest that metabolomic analysis holds promise as a diagnostic tool for distinguishing these disorders. While veterans with GWI have higher rates of CFS [72], the characteristics of CFS in GWI differ from ME/CFS in civilians [5, 73]. Future studies will be required to specifically compare the metabolomic signature of CFS concurrent with GWI to ME/CFS in civilians. Just as there are veterans with GWI who have symptoms compatible with CFS, so there may be civilians with ME/CFS who have had similar chemical or biological exposures encountered outside of military service, but express a chemical phenotype similar to GWI. Chronic fatiguing illness has been reported after non-military exposure to organophosphate pesticides [86]. Organophosphate pesticides are inhibitors of acetylcholinesterase. Exposures to acetylcholinesterase inhibitors, including organophosphate pesticides themselves [87], occurred in the Gulf, and have been implicated in GWI [5]. Chronic fatigue resulting from civilian organophosphate exposure was reportedly more similar to CFS in veterans with GWI than in other civilians with ME/CFS [88]. Metabolomics may clarify additional similarities and differences of organophosphate-associated fatigue relative to GWI and non-organophosphate associated ME/CFS. In both GWI and CFS, metabolomics underscored the involvement of mitochondria.

These data suggest several testable hypotheses with treatment implications, and may revise our understanding of the mechanisms of existing treatments. For instance, coenzyme Q10 (coQ10), reported to significantly improve symptoms and function in veterans with GWI [89], is known to support electron transport in mitochondria, inhibit redox changes under cell stress, and protect the cell against free radical injury. Cell culture experiments have demonstrated that coQ10 can also prevent apoptosis by decreasing ceramide release [90], and both *in vivo* and *in vitro* studies showed that coQ10 can decrease apoptosis in response to ceramide exposure [91].

The prominence of purine and pyrimidine metabolism in the signature of GWI (Table 3, Fig 1B), the importance of purinergic signaling in the molecular stages of healing [66], and the adaptation of energy use to energy availability, suggest that therapies directed at purinergic signaling might constitute a fresh approach to treatment [66, 77]. Similarly, the reductions of measured endocannabinoids may support an appraisal of cannabinoid therapy. With regard to potential surrogate markers of disease, the robust increase in nearly a dozen plasma ceramides in GWI veterans suggests that a reduction in ceramides might merit assessment as a biochemical marker of treatment benefit in new clinical trials.

## Conclusions

The metabolomic signature of GWI was dominated by increases in ceramides, sphingomyelins, and phosphatidylcholine lipids, and a decrease in plasma purines (Figs 1, 2, 3 and 4, Tables 2 and 3, S1 Table, S1, S2 and S3 Figs). These differences offer novel insights into the underlying biology and have implications for new approaches to treatment. Metabolomic findings in this human study are buttressed by concordant findings in mouse and rat models of GWI. Future studies should encompass geographically distinct samples, larger sample numbers, include a measure of clinical severity, and extend assessment to female veterans.

## Supporting information

S1 Table Discriminating metabolites. (XLSX)

S2 Table Acylcarnitines. (XLSX)

S3 Table Pairwise spearman correlations of metabolite Z-scores in veterans with Gulf War illness. (XLSX)

S4 Table Pairwise spearman correlations of metabolite Z-scores in controls. (XLSX)

S5 Table GWI metabolomics raw AUC data. (CSV)

S1 Fig Metabolic map of pathways disturbed in Gulf War illness. Colored circles reflect metabolite changes measured as Z-scores. Red shaded metabolites were increased, green shaded metabolites were decreased in GWI. Yellow circles were measured but found to be unchanged. White circles reflect metabolites that were not measured in this study.
(PDF)

S2 Fig Principal components analysis. **A**. Scree Plot. The green line represents the cumulative variance explained by each added component. The blue line represents the individual percent variance explained by each component. **B**. Top 5 Positive and Negative Factors in PCA Components 1 and 2.
(PDF)

S3 Fig Metabolomic networks in Gulf War illness. **A**. Gulf War illness, **B**. Controls. Gray colored connectors represent positive correlations. Black colored connectors represent negative correlations. N = 20 cases and 20 controls. 358 plasma metabolites were measured. Same-colored metabolites in each subnetwork are from the same biochemical pathway. Only correlations with a Pearson r value ≥ 0.85 are shown.
(PDF)
