# Supplementary figures and images for: Metabolic features of Gulf War illness
Source: PLoS One. 2019 Jul 26;14(7):e0219531. doi: 10.1371/journal.pone.0219531 (PMC6660083; doi:10.1371/journal.pone.0219531)

# Figure S1

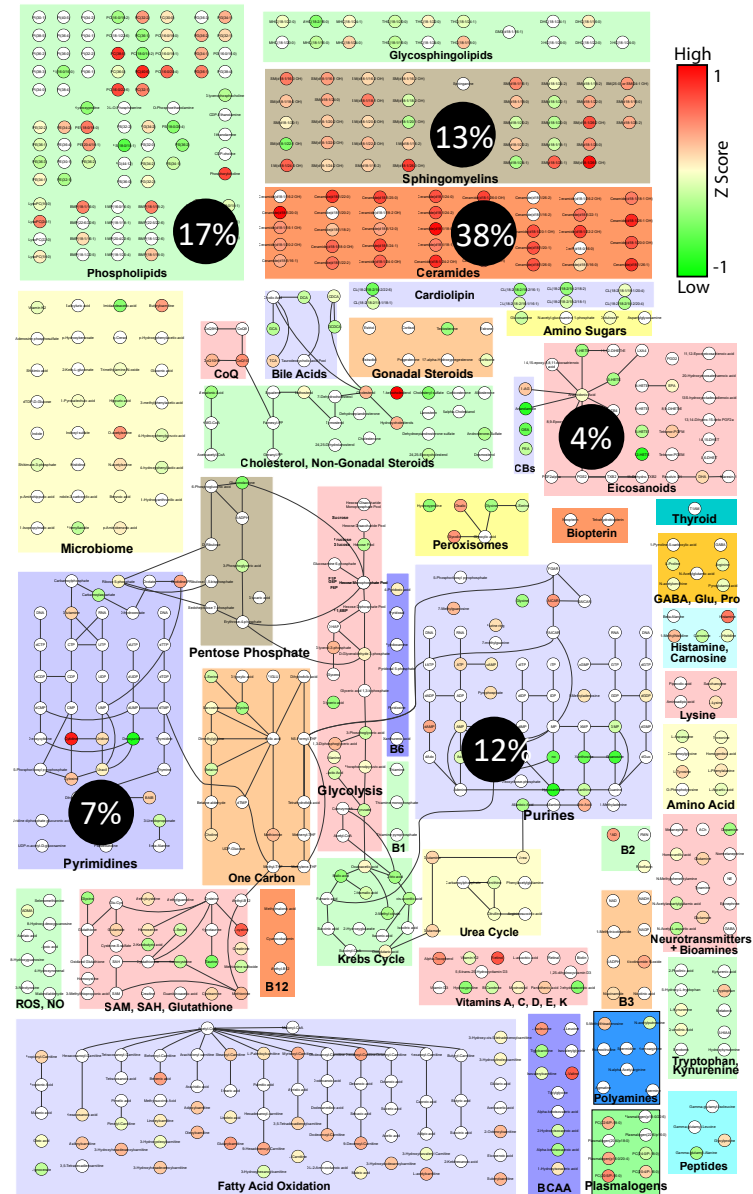

Supplement: S1 Fig — Colored circles reflect metabolite changes measured as Z-scores. Red shaded metabolites were increased, green shaded metabolites were decreased in GWI. Yellow circles were measured but found to be unchanged. White circles reflect metabolites that were not measured in this study. (PDF) [file pone.0219531.s006.pdf]

# Figure S3.

**A**

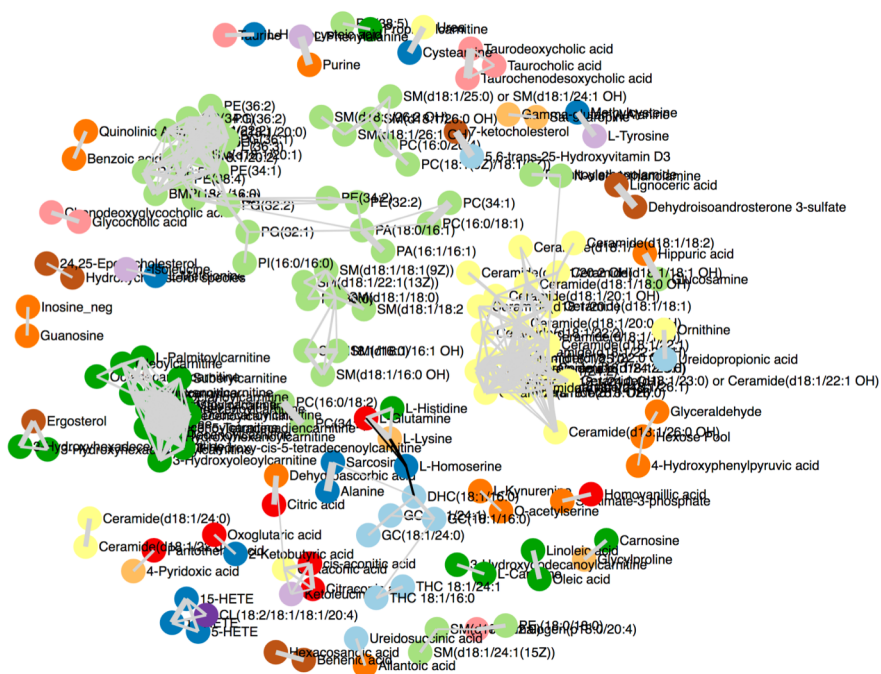

# B

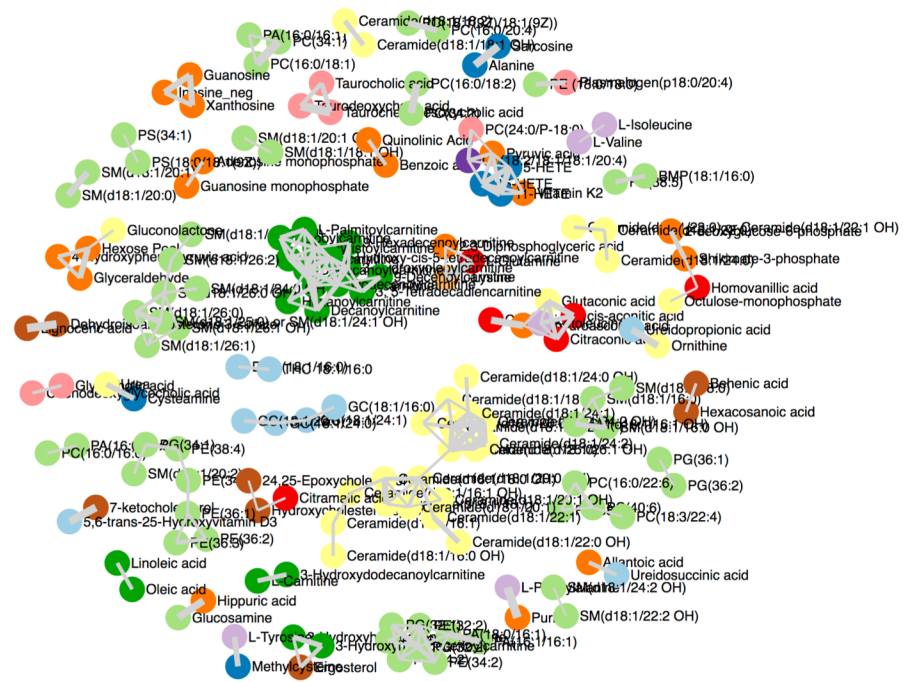

Supplement: S3 Fig — A. Gulf War illness, B. Controls. Gray colored connectors represent positive correlations. Black colored connectors represent negative correlations. N = 20 cases and 20 controls. 358 plasma metabolites were measured. Same-colored metabolites in each subnetwork are from the same biochemical pathway. Only correlations with a Pearson r value ≥ 0.85 are shown. (PDF) [file pone.0219531.s008.pdf]
