# Supplementary material for: Metabolic features of Gulf War illness
Source: PLoS One. 2019 Jul 26;14(7):e0219531. doi: 10.1371/journal.pone.0219531 (PMC6660083; doi:10.1371/journal.pone.0219531)
Supplement: S2 Fig — A. Scree Plot. The green line represents the cumulative variance explained by each added component. The blue line represents the individual percent variance explained by each component. B. Top 5 Positive and Negative Factors in PCA Components 1 and 2. (PDF) [file pone.0219531.s007.pdf]

Figure S2

A

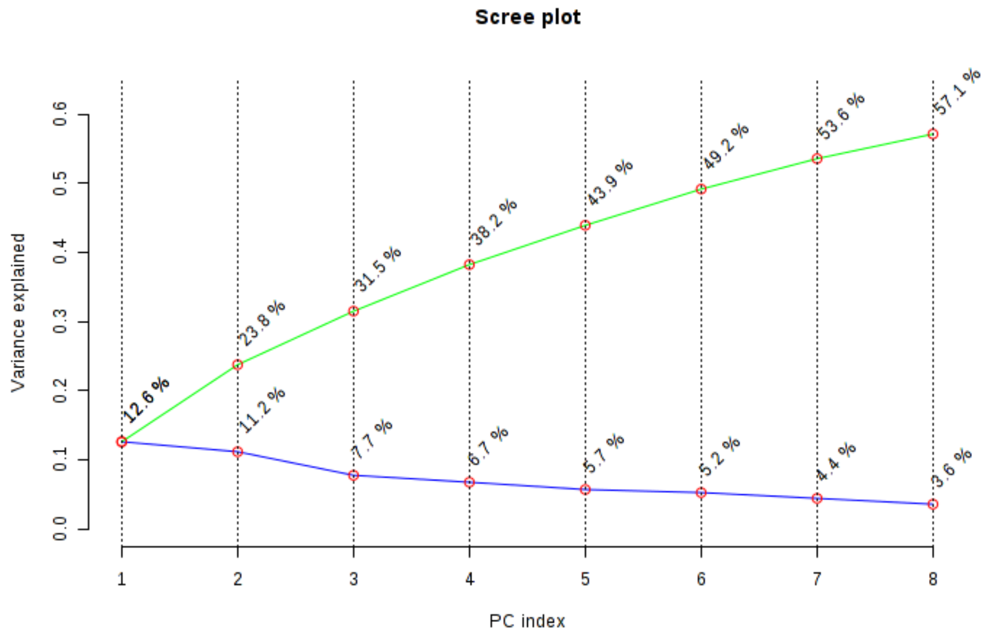

B

No.	PC1 Metabolite	PC1 (12.6%)	No.	PC2 Metabolite	PC2 (11.2%)
1	Ceramide(d18:1/18:0 OH)	0.11454	1	L-Homoserine	0.11309
2	Ceramide(d18:1/20:0 OH)	0.1134	2	Pimelylcarnitine	0.10365
3	Ceramide(d18:1/18:1)	0.11268	3	Octadecanoylcarnitine	0.10112
4	Ceramide(d18:1/24:2 OH)	0.11231	4	L-Palmitoylcarnitine	0.10024
5	Ceramide(d18:1/20:1 OH)	0.11229	5	Adipoylcarnitine	0.096754
6	Dehydroisoandrosterone 3-sulfate	-0.055086	6	THC(18:1/18:0)	-0.1009
7	Cortisone	-0.056052	7	L-Proline	-0.10535
8	LysoPC(22:0)	-0.05668	8	GC(18:1/16:0)	-0.10621
9	Inosine	-0.058591	9	THC (18:1/16:0)	-0.1105
10	PC(20:4/P-16:0)	-0.058668	10	DHC(18:1/16:0)	-0.12021
